# A 25K Wheat SNP Array Revealed the Genetic Diversity and Population Structure of Durum Wheat (*Triticum turgidum* subsp. *durum*) Landraces and Cultivars

**DOI:** 10.3390/ijms26157220

**Published:** 2025-07-25

**Authors:** Lalise Ararsa, Behailu Mulugeta, Endashaw Bekele, Negash Geleta, Kibrom B. Abreha, Mulatu Geleta

**Affiliations:** 1 Department of Microbial Cellular and Molecular Biology, College of Natural Sciences, Addis Ababa University, Addis Ababa P.O. Box 1176, Ethiopia; lalise.arasa@aau.edu.et (L.A.); endashawbw@gmail.com (E.B.); 2 Department of Plant Breeding, Swedish University of Agricultural Sciences, 234 56 Alnarp, Sweden; behailu.mulugeta@slu.se (B.M.); kibrom.abreha@slu.se (K.B.A.); 3 Department of Plant Science, Salale University, Fiche P.O. Box 245, Ethiopia; 4 Sinana Agricultural Research Center, OARI, Bale-Robe P.O. Box 208, Ethiopia; 5 Kulumsa Agricultural Research Center, Ethiopian Institute of Agricultural Research, Assela P.O. Box 489, Ethiopia; negash.geleta2020@gmail.com

**Keywords:** durum wheat genomics, population structure, landrace conservation, selection signatures, single nucleotide polymorphism (SNP)-based genotyping

## Abstract

Durum wheat, the world’s second most cultivated wheat species, is a staple crop, critical for global food security, including in Ethiopia where it serves as a center of diversity. However, climate change and genetic erosion threaten its genetic resources, necessitating genomic studies to support conservation and breeding efforts. This study characterized genome-wide diversity, population structure (STRUCTURE, principal coordinate analysis (PCoA), neighbor-joining trees, analysis of molecular variance (AMOVA)), and selection signatures (F_ST_, Hardy–Weinberg deviations) in Ethiopian durum wheat by analyzing 376 genotypes (148 accessions) using an Illumina Infinium 25K single nucleotide polymorphism (SNP) array. A set of 7842 high-quality SNPs enabled the assessments, comparing landraces with cultivars and breeding populations. Results revealed moderate genetic diversity (mean polymorphism information content (PIC) = 0.17; gene diversity = 0.20) and identified 26 loci under selection, associated with key traits like grain yield, stress tolerance, and disease resistance. AMOVA revealed 80.1% variation among accessions, with no significant differentiation by altitude, region, or spike density. Landraces formed distinct clusters, harboring unique alleles, while admixture suggested gene flow via informal seed exchange. The findings highlight Ethiopia’s rich durum wheat diversity, emphasizing landraces as reservoirs of adaptive alleles for breeding. This study provides genomic insights to guide conservation and the development of climate-resilient cultivars, supporting sustainable wheat production globally.

## 1. Introduction

Durum wheat (*Triticum turgidum* subsp. *durum*) is a globally significant cereal crop and the primary raw material for pasta, couscous, and other semolina-based products. With an annual production of approximately 40 million metric tons [1,2], it is the second most cultivated wheat species after bread wheat (*Triticum aestivum*). It plays a critical role in the Mediterranean basin, North America, and parts of Sub-Saharan Africa, where it serves as a dietary staple and supports smallholder farmers’ livelihoods [3,4]. However, its production is increasingly constrained by climate change-induced abiotic stresses (e.g., drought and heat), evolving pathogens, and the narrowing genetic diversity of modern high-yielding cultivars [5]. These challenges pose significant risks to global food security, particularly in regions where durum wheat is a cornerstone of traditional diets and agricultural systems [4].

Genetic diversity is fundamental for crop improvement, providing the necessary variation to develop varieties with enhanced yield, stress tolerance, and nutritional quality [6]. In contrast, modern breeding programs frequently rely on a limited pool of elite germplasm, which reduces genetic diversity and may result in the loss of valuable alleles [7]. The landraces and wild relatives of durum wheat, on the other hand, contain genetic variations that have been shaped by centuries of natural selection and farmer-mediated selection. Ethiopia, recognized as a center of diversity for tetraploid wheat [8,9], hosts an exceptional array of durum wheat landraces cultivated across diverse agroclimatic zones spanning 1500–3000 m above sea level [10]. These landraces exhibit substantial variation in desirable traits, including drought resilience, disease resistance, and grain quality [11,12,13,14,15]. Despite this potential, there has been a decline in durum wheat cultivation in Ethiopia due to an expansion in the cultivation of bread wheat [10,16]. This shift risks genetic erosion and the loss of locally adapted alleles critical for climate-resilient breeding [17].

In recent years, high-throughput genotyping, next-generation sequencing, and DNA chip technology have revolutionized crop genetic diversity research, making it possible to identify genomic regions under selection, dissect population structure, and identify marker–trait associations [18,19,20]. SNP arrays, such as Infinium 25K wheat SNP arrays [21], allow genome-wide marker analysis at a cost-effective price, which facilitates breakthroughs in understanding the genetic architecture of complex traits, the detection of selective sweeps associated with domestication and adaptation, and the identification of novel alleles for breeding [22].

Most studies on Ethiopian durum wheat diversity have focused on phenotypic traits, revealing high morphological variation [23,24,25]. In recent studies, genome-wide single-nucleotide polymorphism (SNP) markers have been used to analyze genetic diversity in landraces and cultivars [9,26,27,28,29]. However, many of these studies either used a single genotype per accession [9,28] or pooled samples for sequencing [26,27], limiting insights into within-accession genetic variation. Additionally, although the Ethiopian Biodiversity Institute (EBI) maintains extensive durum wheat collections, most accessions remain uncharacterized at the genomic level, limiting their potential use in breeding programs.

With climate change and genetic erosion posing threats, comprehensive genomic studies are essential, which prompts this high-resolution analysis of Ethiopian durum wheat genetic resources to gain a deeper understanding of their potential and facilitate their conservation and use in modern breeding programs. This study aimed to (1) assess genome-wide diversity and population structure to elucidate genetic relationships, (2) identify signatures of selection and detect genes under selection, (3) facilitate the development of genomic resources for marker-assisted breeding, and (4) evaluate within-population variation in landraces compared to improved materials to optimize genotyping strategies for marker–trait association studies. 

## 2. Results

In the present study, 376 durum wheat genotypes were genotyped with an Illumina Infinium 25K wheat SNP array containing 24,146 SNPs. Of these, 14.2% (3426 SNPs) failed, and 7367 (35.5%) of the remaining 20,720 SNPs had missing values. Of the remaining 13,353 SNPs without missing data, 5511 SNPs (41.2%) were monomorphic across the 376 genotypes while 7842 SNPs (58.7%) were polymorphic with two alleles. In total, 6744 (86%) of the 7842 polymorphic SNPs with no missing data were accurately mapped to the 14 chromosomes of the wheat A and B genomes. The number of markers mapped to each chromosome ranged from 270 (Chromosome 4B) to 593 (Chromosome 7A), with a total of 3322 and 3342 markers mapped to A and B genomes, respectively (Table 1). The genomic regions covered in each chromosome ranged from 74.3 Mbp (Chromosome 3A) to 836.1 Mbp (Chromosome 3B), with a total of 4176.5 Mbp and 9272.3 Mbp regions covered in A and B genomes, respectively (Table 1).

### 2.1. Genomic-Wide Genetic Variation

**Figure 1:**
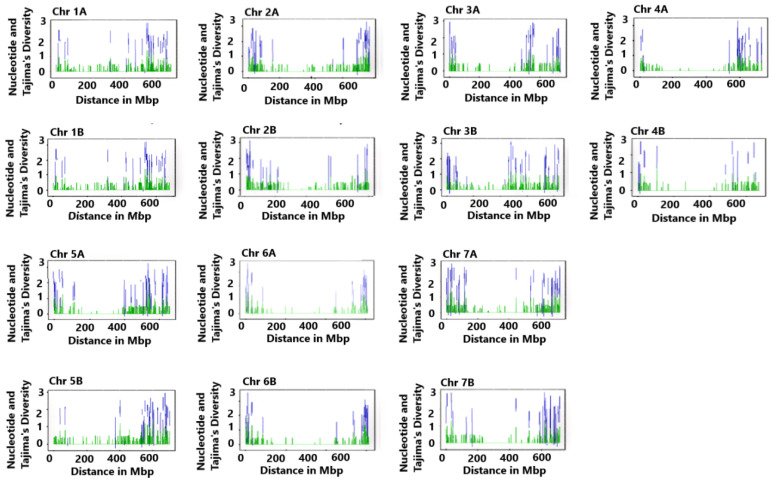
A chromosome level genome diversity pattern of durum wheat accessions used in this study. A sliding window (window size = 1 Mb, step size = 200 kb) was used to analyze nucleotide diversity and Tajma’s D. The green and blue peaks show nucleotide diversity and Tajma’s D, respectively. The overall average nucleotide diversity (π) and Tajma’s D were 0.20 and 1.39, respectively.

Nucleotide diversity and Tajima’s D were used to examine genome-wide variation and selection signatures. At the individual chromosome level, the highest average nucleotide diversity (0.22) and Tajima’s D (1.72) were recorded on chromosome 4B and the lowest on chromosome 2B, which were 0.19 and 1.16, respectively (Table 1). The mean nucleotide diversity of the A and B genomes was 0.21 and 0.20, respectively, with an overall mean of 0.20. The mean Tajima’s D of A and B genomes was 1.41 and 1.38, respectively, with an overall average of 1.40 (Table 1). A notable reduction in nucleotide diversity and Tajima`s D was observed in the pericentromeric regions of the chromosomes, particularly in chromosomes 4A, 4B, 6A, and 6B (Figure 1). In contrast, each chromosome’s distal regions displayed substantial nucleotide diversity and Tajima’s D.

**Figure 2:**
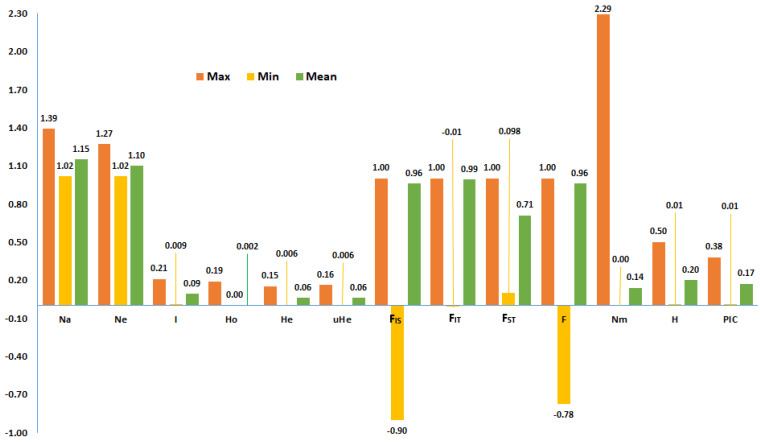
The mean, minimum, and maximum values for the number of alleles (Na), number of effective alleles (Ne), Shannon informative index (I), observed heterozygosity (Ho), expected heterozygosity (He), unbiased expected heterozygosity (uHe), fixation indices (F_IS_, F_IT_, F_ST_, F), polymorphism information content (PIC), and gene diversity (H) for 7842 polymorphic SNP loci distributed across the 14 durum wheat chromosomes.

The mean effective number of alleles (Ne) of the SNP loci over accessions ranged from 1.02 to 1.27, with a grand mean of 1.1, whereas Shannon diversity index (I), observed heterozygosity (Ho), and expected heterozygosity (He) ranged from 0.009 to 0.21, 0.0 to 0.19, and 0.006 to 0.15, with grand means of 0.09, 0.002, and 0.06, respectively (Figure 2). Across all accessions, gene diversity (H) of SNP loci ranged from 0.01 to 0.50 (mean = 0.20), while polymorphism information content (PIC) ranged from 0.01 to 0.38 (mean = 0.17). In the case of fixation indices calculated over all accession for each locus, the minimum, maximum, and mean values were −0.90, 1.00, and 0.88 for F_IS_; −0.01, 1.00, and 0.93 for F_IT_; 0.00, 1.00, and 0.67 for F_ST_; and −0.78, 1.0, and 0.96 for F, respectively. The average gene flow (Nm) per locus was 1.4 (Figure 2).

### 2.2. SNP Loci Under Selection

The analysis of 7842 polymorphic SNPs conducted using the non-hierarchical finite island model, with false discovery rate (FDR) adjustment to control for false positives, revealed that 446 loci were under selection (significant at *p* < 0.01) (Supplementary Table S2). Using the Variant Effect Predictor (VEP) tool available at https://plants.ensembl.org/tools.html (accessed on 3 July 2025), these 446 loci were further analyzed to predict mutation effects. This analysis resulted in 26 genic loci whose SNPs resulted in a stop codon gain or loss, or amino acid change with moderate to high impacts on their proteins as predicted by the VEP tool (Table 2). These SNP loci were distributed across all chromosomes except 3B and 5A. Eleven and fifteen of these loci were on the A and B genomes, respectively. The observed F_ST_ values of these loci varied from 0.58 to 0.98 (F_ST_ *p*-value < 0.01). The mutation type at 18 of these loci were missense mutations with predicted moderate effect on their corresponding proteins, according to their Sorting Intolerant from Tolerant (SIFT) scores (<0.05). The mutation types in the remaining eight loci were stop-gained (five loci) and stop-lost (three loci) (Table 2). Four of the five stop-gained mutations were the results of C/T mutations that changed the glutamine codon (CAG) to a stop codon (TAG), while the fifth one was the result of an A/G mutation that changed the leucine codon (TTA) to a stop codon (TGA). In three stop-lost mutations, glutamic acid, leucine, and serine replaced the stop codons (Table 2).

### 2.3. Genetic Variation Between and Within Accessions and Their Groups

Various population genetic parameters were analyzed for the 57 accessions represented by five genotypes each, based on 7842 polymorphic SNP loci (Table 3). The effective number of alleles (Ne) ranged from 1.0 (22 accessions) to 1.42 (accession 31248), with a mean of 1.05, whereas the mean Shannon’s information index was 0.04, with values ranging from 0.0 (23 accessions) to 0.32 (accession 31248). The observed heterozygosity (Ho) ranged from 0.0 (55 accessions) to 0.03 (accession 31248), with a mean of 0.001, while the expected heterozygosity (He) ranged from 0.0 (24 accessions) to 0.22 (accession 31248), with a mean of 0.03. The fixation index (F) of the accessions spans its complete range of −1.0 (eight accessions) to 1.0 (14 accessions). The percent polymorphic loci (%PL) of the accessions ranged from 0.0 (10 accessions) to 49.1% (31248), with a mean of 7.9%. The number of loci with private alleles ranged from 0.0 (19 accessions) to 1685 (accession 31248). The number of loci with private alleles in accession 31248 accounted for 21.5% of the loci analyzed. On average, 36 loci (0.5% of the loci studied) had private alleles. The mean frequency of private alleles per population varied from 0.2 (five accessions) to 1.0 (12 accessions). The nucleotide diversity (ND) of the accessions ranged from 0.0 (24 accessions) to 0.25 (accession 31248), whereas Tajima’s D (TD) ranged from −2.1 (accession 33523) to 2.6 (accessions 31252, 31146, and 33286). The overall mean Tajima’s D of the accessions was 0.82. Four accessions had negative Tajima’s D (accessions 31209, 33239, 33235, and 33523). Among accessions with negative Tajima’s D, accession 33523 had the lowest value of −2.12 (*p* < 0.001). Tajima’s D of eight accessions whose values ranged from 2.19 to 2.55 had *p*-values below 0.05 (Table 3).

The accessions were grouped into different groups based on various criteria for determining their genetic diversity parameters: administrative regions of origin, altitude of collection sites, accession types, and spike density (Table 4). Among the three administrative regions, accessions from the Oromia region had the highest mean number of loci with private alleles (NLPA = 7). In contrast, the highest mean fixation index (F = 0.60) was recorded for Amhara region accessions. The %PL for Amahara, Oromia, and Tigray were 7.9, 8.0, and 7.5, respectively. The other parameters analyzed were quite similar across the three regions. The grouping of landrace accessions according to their altitudinal range showed that accessions collected at altitudes ranging from 2000 to 2500 m above sea level (masl) had higher He (0.040), %PL (10%), NLPA (12), and Tajima’s D (1.27) (Table 4) compared to the other two altitude ranges (below 2000 masl and above 2500 masl). The fixation index (F) of accessions collected at altitudes below 2000 masl was higher (0.97) than that of the other two altitude ranges. The mean number of loci with private alleles in landraces was higher than that in improved cultivars (12) and breeding populations (17). On the other hand, improved cultivars had higher mean values of He (0.05), F (0.98), and %PL (14.3%) compared to landraces and breeding populations. In the case of spike density, accessions with very dense spikes had higher He (0.038), F (0.67), %PL (9%), and NLPA (66), on average, compared to accessions with dense and lax spikes (Table 4).

### 2.4. Analysis of Molecular Variance (AMOVA) and Population Differentiation

Analysis of molecular variance (AMOVA) of 57 durum wheat accessions revealed that 80.1% of the total genetic variation was among the accessions. Variations within accessions and individuals were 19.3% and 0.6%, respectively (Table 5). The fixation indices F_IS_, F_ST_, and F_IT_ were 0.80, 0.97, and 0.99, respectively, and, together with their corresponding variance components, all of them were highly significant (*p* < 0.001). The hierarchical AMOVA conducted by grouping the 57 accessions into improvement-status groups (landraces, cultivars, and breeding populations) showed that 12.3% of the total variation was found among these groups, which was highly significant (F_CT_ = 0.44; *p* < 0.001). However, no significant variations were found between altitude groups (F_CT_ = −0.05; *p* = 0.93), administrative region groups (F_CT_ = −0.038; *p* = 0.93), and spike density groups (F_CT_ = 0.01; *p* = 0.19) (Table 5).

**Figure 3:**
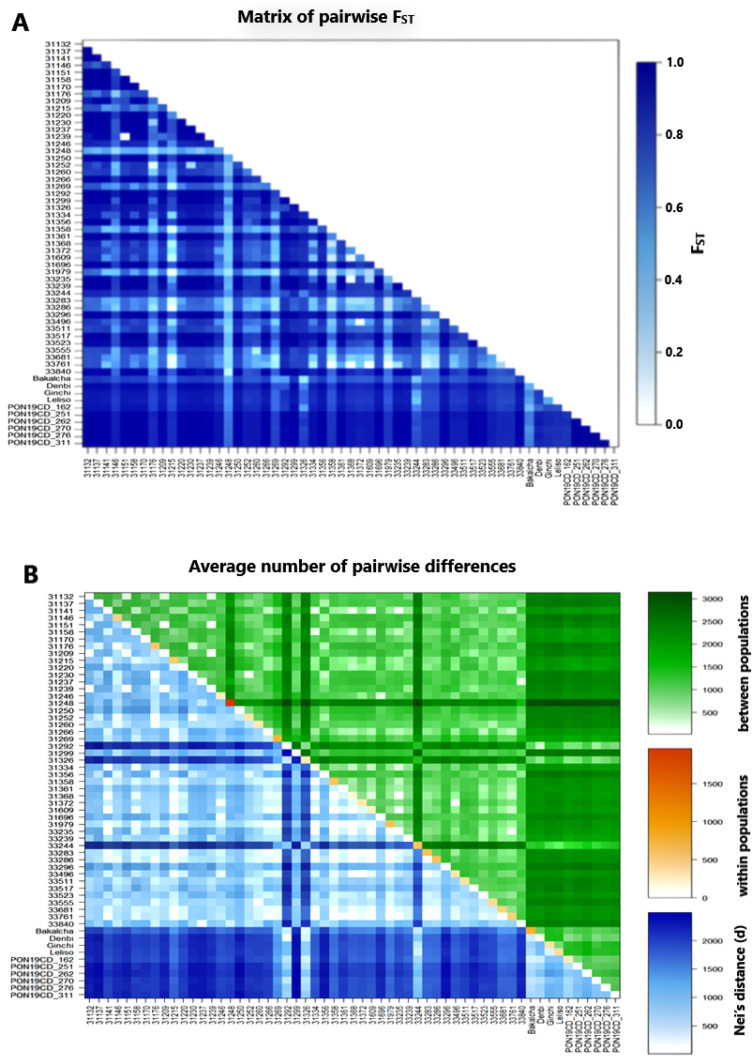
Heatmaps of pairwise genetic differentiation (F_ST_) among 57 durum wheat accessions (**A**) and the average number of pairwise differences within and between 57 durum wheat accessions (**B**). The average number of pairwise differences was estimated using the number of different alleles as a distance method, where the heatmaps of pairwise differences among the accessions (PiXY), pairwise differences within the accession (PiX), and corrected average pairwise differences (PiXY − (PiX + PiY)/2, also called Nei’s distance (d)) are displayed above the diagonal, diagonally, and below the diagonal, respectively.

**Figure 4:**
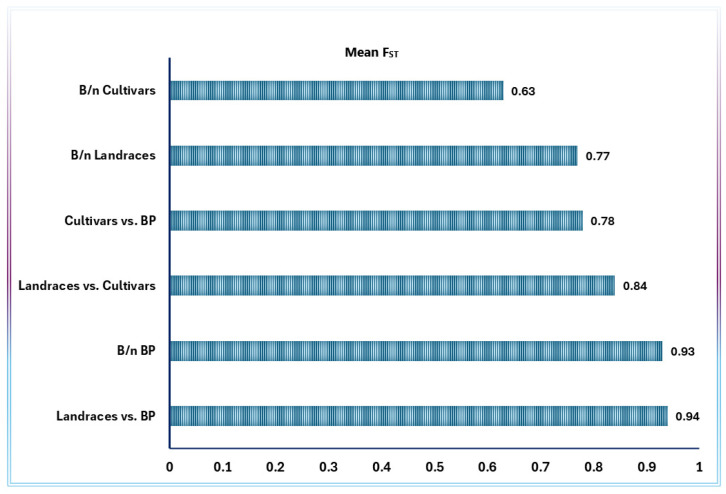
Mean F_ST_ between accessions of breeding populations, between accessions of cultivars, and between accessions of landraces, as well as between cultivars and breeding populations, between cultivars and landraces, and between breeding populations and landraces. BP = breeding populations; b/*n* = between. F_ST_ (fixation index) measures genetic differentiation among sub-populations (accessions), with higher values indicating greater divergence.

The pairwise genetic differentiation analysis involving 57 accessions, represented by five genotypes each, revealed F_ST_ values ranging from 0.11 to 1.00, with an overall mean F_ST_ of 0.80 (Figure 3A and Supplementary Table S4a–c). Among the 1596 pairwise F_ST_ of the 57 accessions, only 24 (1.5%) were statistically insignificant (*p* > 0.05; Supplementary Table S4a–c). The lowest F_ST_ value of 0.11 was found for accessions 31151 vs. 31239, 31368 vs. 33235, and 31609 vs. 33761 (Supplementary Table S4a). Interestingly, the F_ST_ between each pair of landrace accessions 31158, 31220, 31356, 31361, and 33296 was 1.00, indicating full genetic differentiation between them. The mean F_ST_ of each accession from all other accessions ranged from 0.52 (accession 31248) to 0.93 (accessions PON19CD_251, PON19CD_262, and PON19CD_276) (Supplementary Table S4a). The mean F_ST_ values within breeding populations, cultivars, and landraces were 0.93, 0.63, and 0.77, respectively (Figure 4). On the other hand, the mean F_ST_ values of cultivars versus breeding populations, landraces versus cultivars, and landraces versus breeding populations were 0.78, 0.84, and 0.94, respectively (Figure 4).

The average number of pairwise differences between accessions ranged from 25.8 (between accessions 31151 and 31239) to 3142.9 (between accessions 33244 and 31248), with an overall average of 1480.1 (Figure 3B; Supplementary Table S4d–f). The lowest and highest mean values of average pairwise differences were recorded in accessions 33235 (1175.1) and 31248 (2306.0), respectively. Among the cultivars and breeding populations, the highest mean values of average pairwise differences were recorded in accession PON19CD_262 (2040.1) and Ginchi (2069.4), respectively. Corrected pairwise differences (Nei’s distance) between accessions were also calculated (Figure 3B; Supplementary Table S4d–f), which ranged from 2.7 (between accessions 31151 and 31239) to 2497.7 (between accessions 31299 and PON19CD_311). The lowest and highest mean Nei’s distance was recorded in accessions 31979 (789.0) and PON19CD_262 (188.8), respectively. Among landrace accessions, accession 31292 had the highest mean Nei’s distance (1757.9). The average number of pairwise differences within accession ranged from zero (accessions 31158, 31220, 31356, 31361, and 33296) to 1959.4 (accession 31248), with an overall mean of 794.7 (Figure 3B; Supplementary Table S4d–f).

### 2.5. Cluster Analysis

**Figure 5:**
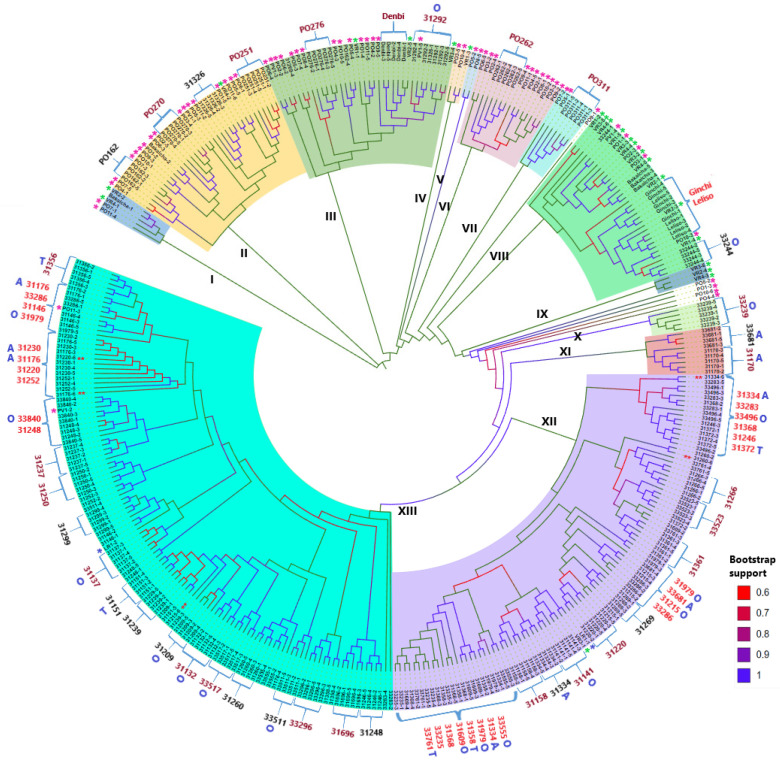
Ne’s unbiased genetic distance-based neighbor-joining tree of 376 genotypes from 148 durum wheat accessions (57 accessions were represented by five genotypes each while 91 accessions were represented by a single genotype each). Accessions represented by single plants are shown with an asterisk on the outer side (pink, green, and blue asterisks refer to breeding populations, cultivars, and landraces, respectively). A double red asterisk on the inner side indicates genotypes that differ in spike morphology from other genotypes within the same accessions. Accessions whose five genotypes were most closely clustered together are shown in dark-red font. Accessions whose four of the five genotypes were most tightly clustered together are given in black font. Sub-clusters containing genotypes from multiple accessions are in red font. Capital letters in blue font on the outermost side refer to the administrative regions of origin of landrace accessions (A = Amhara; O = Oromia; and T = Tigray). The administrative regions of origin of unmarked landrace accessions are unknown (see Supplementary Table S1). The colors of the branches indicate their bootstrap support, as shown in the key.

Ne’s unbiased genetic distance-based neighbor-joining cluster analysis of 376 genotypes from 148 durum wheat accessions (57 of which were represented by five genotypes each and 91 of which were represented by a single genotype each) resulted in six major clusters (Clusters II, III, VI, VIII, XII, and XIII) comprising more than 10 genotypes, 7 minor clusters (Clusters I, IV, V, VII, IX, X, and XI) comprising up to 9 genotypes, and 5 solitary genotypes (Figure 5). Cluster I is the most divergent cluster, comprising five genotypes from breeding populations and cultivars. Cluster II included 36 genotypes, most of which represent breeding populations. However, this cluster also contained a landrace accession (accession 31326), the most divergent accession among landraces. Similarly, Cluster III contained 36 genotypes, most of which were from breeding populations. In addition, this cluster included cultivar Dendi and landrace accession 31292. All genotypes in Clusters IV (three accessions), V (two accessions), VI (16 accessions), and VII (seven accessions) were from breeding populations, except a single genotype (VR1-3) representing a cultivar. Cluster VIII included 34 genotypes, most of which were from cultivars. Landrace accession 33244 was the only landrace accession in this cluster (Figure 5). Cluster IX had only three genotypes from cultivars. Clusters X (five genotypes), XI (nine genotypes), Cluster XII (96 genotypes), and XIII (119 genotypes) comprised landrace genotypes, except for one genotype in Cluster XII and two genotypes in Cluster XIII representing cultivar and breeding populations, respectively.

Among cultivars represented by five genotypes each, the most diverse was Bakalcha, with its genotypes distributed in Clusters I, II, and VIII. Five genotypes of each breeding population were clustered together, except for one genotype of PO162, which was separated from the other four genotypes in Cluster II and placed in Cluster III. Among the 47 landrace accessions with five genotypes each, 17 accessions had all genotypes clustered tightly together. Four of the five genotypes in 12 landrace accessions were tightly clustered together, while one genotype of each accession was in a different branch within the same cluster or in a different cluster. For example, four genotypes from accession 31326 were tightly clustered in Cluster II, while the fifth genotype was clustered with genotypes from accession 31292. Meanwhile, genotypes of 18 landrace accessions were intermixed across various branches of Clusters XII and XIII. Among these, genotypes of accession 33286 formed two groups, three of which were clustered in Cluster XII while the other two were clustered in Cluster XIII (Figure 5). Similarly, four genotypes of accession 31979 were distributed across two branches of Cluster XII while the fifth genotype was placed in Cluster XIII. A noteworthy observation is that genotypes that differed in spike morphology from other genotypes of the same accessions were almost always placed separately from the other genotypes. Another interesting point was that landrace accessions did not show a clear clustering pattern according to their administrative region of origin. For example, Cluster XII’s last branch included 23 genotypes from eight accessions representing all three administrative regions (Amhara, Oromia, and Tigray) (Table 5).

### 2.6. Principal Coordinate Analysis and Population Structure

**Figure 6:**
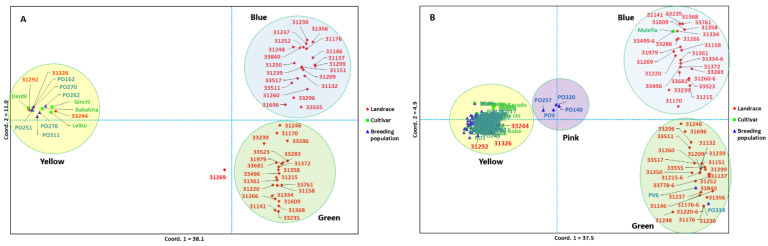
Principal coordinate analysis (PCoA) of (**A**) 57 durum wheat accessions represented by five genotypes each and (**B**) 148 accessions represented by one genotype each. Different font colors and symbols represent different accession types: landraces, cultivars, and breeding populations. Note: to generate the second PCoA (**B**), one genotype was randomly sampled from each of the 57 accessions originally represented by five genotypes.

Principal coordinate analysis (PCoA) was performed to determine the relationship between the 57 accessions represented by five genotypes each (Figure 6A) as well as between 148 accessions, where each accession was represented by a single genotype (Figure 6B). For the 57 accessions, the first two principal coordinates (PCos) explained 49.1% of the total variation, with PCo1 explaining 38.1% (Figure 6A). In this analysis, accessions formed three clusters, with the yellow cluster distinctly separated from the green and blue clusters along PCo1. The green and blue clusters were separated along PCo2. All four cultivars and six breeding populations were clustered in the yellow cluster, which also contained three landrace accessions (31292, 31326, and 33244). Another landrace accession (31269) was separated from the other accessions along PCo1. All accessions in the green and blue clusters were landrace accessions. The clustering pattern of the 148 accessions was similar to that of the 57 accessions, although they formed four clusters. Here, PCo1 and PCo2 accounted for 37.5% and 4.9%, respectively. The yellow cluster is the most distinct group, distinguished from the other clusters along PCo1. It contained most breeding populations, all cultivars except one, and three landrace accessions (31292, 31326, and 33244). All landrace accessions (except the four aforementioned ones) were clustered in green and blue clusters that were separated along PCo2. Malefia was the only cultivar separated from the other cultivars and placed in the blue cluster. In addition to landrace accessions, the green cluster contained two breeding populations (PV6 and PO333) (Figure 6B).

**Figure 7:**
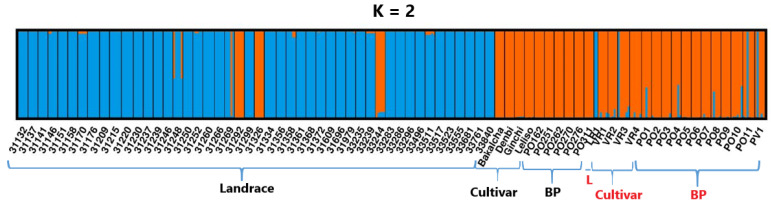
Graphical display of the optimal genetic structure of the 376 genotypes representing the 148 durum wheat accessions. Blue and deep orange graph colors represent the two clusters (K) identified in the population structure analysis. The first 57 bars correspond to 57 accessions represented by five genotypes each (47 landraces, 4 cultivars, and 6 breeding populations, in that order). The last 17 bars represent 91 single-genotype accessions grouped into 17 groups for visualization: 1 landrace (LR1), 4 cultivars (VR1-VR4), and 12 breeding populations (PO1-PO11, PV1). L = Landrace, BP = Breeding population.

The admixture model-based population structure analyzed using STRUCTURE [30] and STRUCTURESELECTOR programs [31] revealed that the optimal number of genetic populations (K) defining the 376 genotypes from 148 accessions is two, as per the delta K method of Evanno et al. [32] (Figure 7). At K = 2, the graphical representation of the population structure of the accessions demonstrated that most accessions fully belong to either of the two genetic populations. The vast majority of landrace accessions belong to the blue genetic population, with some accessions showing a low level of admixture from the orange genetic population. In contrast, landrace accessions 31292, 31326, and 33244 belong to the orange genetic population. Landrace accessions 31248 and 31269 had a relatively higher level of admixture among the 57 accessions represented by five genotypes each (Figure 7).

## 3. Discussion

The analysis of genetic variation in crops facilitates the enhancement of crop productivity, nutritional quality, and resistance or tolerance to biotic and abiotic stresses through targeted breeding efforts. A critical first step in any plant breeding program involves expanding the genetic diversity of breeding populations, thereby strengthening their potential for crop improvement. This study provides a detailed analysis of genetic diversity, population structure, and selection signatures in durum wheat based on a diverse panel of 376 genotypes genotyped using an Illumina Infinium 25K SNP array. The findings offer significant insights into the genomic architecture, evolutionary forces, and differentiation among Ethiopian durum wheat accessions, with implications for breeding and conservation.

### 3.1. Genome-Wide SNP Distribution and Genetic Diversity

The Illumina Infinium 25K wheat SNP array used in this study was specifically designed for durum wheat (AABB), with markers optimized for the A and B genomes. After quality filtering, 13,353 SNPs were retained for analysis, of which 7842 (58.7%) were polymorphic across the 376 genotypes. The remaining 5511 (41.3%) SNPs were monomorphic, despite including diverse germplasm comprising local landraces, improved varieties, and exotic breeding materials. This moderate polymorphism rate, consistent with findings by Mulugeta et al. [33], suggests that while the 25K array is valuable for assessing broad genetic diversity, it may have limitations in detecting finer-scale variation within Ethiopian durum wheat germplasm. The array enabled comprehensive genome-wide analysis, with 6744 polymorphic SNPs mapped across the A and B genomes. Marker distribution showed positive correlation with chromosome sizes, with the B genome containing slightly more markers than the A genome, reflecting their relative physical sizes. Chromosome 7A contained the highest number of markers (593), while chromosome 4B had the fewest (270), a pattern similar to (though not identical) with Mulugeta et al.’s observations [33].

While the 25K array remains a practical tool for diversity assessments and marker–trait association studies, higher-density platforms such as the 90K array or genotyping-by-sequencing (GBS) could provide enhanced resolution for breeding applications requiring greater marker density, such as genomic selection. These alternatives may offer improved detection of population-specific variants and better coverage of genomic regions currently underrepresented in the 25K array.

The mean effective number of alleles (Ne) of 1.1 indicates that the final SNP set used for genetic analysis in this study contains a high proportion of low-frequency minor alleles, whereas the extremely low mean observed heterozygosity (Ho) of 0.002 is consistent with durum wheat’s inbreeding reproductive mechanism [5,34,35]. The mean Shannon diversity index (I) of 0.09 and expected heterozygosity (He) of 0.06 suggest low genetic diversity in each studied durum wheat accession across most SNP loci. On the other hand, considering all accessions together (total population), the mean gene diversity (H) of 0.20 indicates moderately high genetic diversity across most loci, although the mean gene diversity estimate for each accession was low. The moderate mean polymorphism information content (PIC) of 0.17 indicates that most SNPs in the marker set have a high level of information. In the study of landraces and cultivars grown in Ethiopia using the same SNP array, Mulugeta et al. [29] reported a mean PIC of 0.27, which was calculated by excluding loci with a minimum allele frequency (MAF) value of below 5%. On the other hand, the mean PIC calculated by including rare alleles for global durum wheat collection genotyped using 35K Affymetrix Axiom wheat breeders’ array was 0.12 [9]. Hence, the SNP array used in the present study is highly suitable for durum wheat genotyping for applications such as population genetics and marker–trait association analysis, as it contains a marker set with genome-wide coverage.

F-statistics, comprising fixation indices (F_IT_, F_IS_, and F_ST_), measure the extent of inbreeding based on the genotypic composition of the total population (T), subpopulations (S), and individuals (I) for each locus [36,37,38]. F_IT_ and F_IS_ values range from −1 to 1 with a value of −1 indicating 100% heterozygosity and a value of 1 indicating 100% homozygosity in each subpopulation. F_ST_, whose values range from 0 to 1, measures the differentiation of subpopulations. It reaches its maximum value when subpopulations are fixed for different alleles. This study considered that the combination of the 57 accessions constitutes the total population, and each accession is a subpopulation.

The grand mean F_IS_ (0.88) and F_IT_ (0.93) values are close to their maximum values of 1, indicating a low number of SNP loci with excess heterozygosity. Since durum wheat is predominantly self-pollinating [5], it is interesting to observe loci with excess heterozygosity in landrace populations. It may suggest recent gene flow from other genetically distinct populations in the form of cross-pollination [34] or selection that favors heterozygosity (heterozygote advantage). The former is most likely the case when a population contains heterozygous individuals across most polymorphic loci, while the latter is most likely the case when excess heterozygosity is observed across multiple populations at the same loci. It could also be due to selection against individuals homozygous for unfavorable alleles.

Tajima’s D values are influenced by both selection and demographic history. While values below negative two often suggest directional selection (excess of rare alleles) and values above two may indicate balancing selection (intermediate-frequency alleles) [39], these interpretations require caution in self-pollinating crops like wheat. Population structure (e.g., subdivision or admixture) and reduced effective recombination can inflate Tajima’s D, mimicking balancing selection [40]. In our study, the mean Tajima’s D (~1.4) falls below the threshold of two but remains positive, consistent with demographic effects such as bottlenecks or substructure, as reported in wheat [41]. Nonetheless, the higher D values in distal chromosomal regions (>600 Mbp) could reflect localized balancing selection or regions of conserved diversity due to functional importance.

Chromosomes 7A, 5A, and 7B have more regions with negative Tajima’s D (Figure 1), indicating the presence of rare alleles in genes subjected to directional selection. Interestingly, most chromosomes had Tajima’s D values of above two at the bottom distal regions (> 600 Mbp) than at the top distal regions (<200 Mbp). This suggests that more genes in the bottom than in the top distal regions could be under balancing selection. Based on a comparison of the top distal regions, chromosome 7A ranked highest in terms of the number of loci with Tajima’s D greater than two. On chromosomes 3B and 1B, loci with Tajima’s D above two covered wider regions than on other chromosomes.

The SNPs identified as under selection in this study are in the bottom distal regions of each chromosome, as can be seen from their SNP positions (Table 1, Supplementary Table S2), suggesting that the region is gene rich. In all chromosomes, nucleotide diversity was higher in distal regions than in central regions. However, chromosomes 4A, 4B, 6A, and 6B had wider chromosomal regions with lower nucleotide diversity than the other chromosomes. Variation in marker density across chromosomes and the recurrence of these diversity patterns in independent studies [26,42] indicate that reduced pericentromeric diversity stems from selective constraints rather than uneven marker distribution. The nucleotide diversity of A and B genomes obtained in this study is similar, as also previously reported [26], indicating that both have undergone similar evolutionary processes. It is interesting to note that Tajima’s D and nucleotide diversity values correspond well to the distribution of protein-coding genes and genetic variation illustrated in Ensembl Plants’ summary of durum wheat chromosomes (https://plants.ensembl.org/Triticum_turgidum/Location/Genome, accessed on 3 July 2025). Overall, a nucleotide diversity of 0.20 and Tajima’s D of 1.40 suggest moderate genetic diversity with an excess of intermediate-frequency alleles, possibly due to balancing selection or population structure [39]. The reduction in diversity in pericentromeric regions supports the hypothesis that these regions experience stronger selective constraints and lower recombination rates, as observed in tetraploid wheat [5,42].

### 3.2. Selection Signatures and Functional Implications

The analysis conducted to identify loci under selection revealed that SNPs at 26 loci resulted in stop codon gains or losses or amino acid changes with moderate to high impact on proteins, as predicted by the VEP tool. Eight of these loci exhibited stop gains or stop losses with predicted high impacts on the corresponding proteins (Table 2), which are discussed below.

The *TRITD2Av1G282370* gene in durum wheat encodes a putative exocyst complex component. TRITD2Av1G282370′s specific functional characterization in durum wheat has not yet been documented. However, its annotation as an exocyst complex component suggests its involvement in plant development and stress responses. By regulating vesicle trafficking essential to cell wall synthesis, nutrient transport, and stress tolerance, this enzyme likely contributes to seed development by facilitating the delivery of stress-responsive proteins and membrane components to the cell surface [43,44,45]. The *TRITD7Av1G274730* gene is annotated as coding for a DNA helicase in durum wheat, which suggests it likely contributes to genome integrity by facilitating DNA replication and repair, stress tolerance by helping the plant cope with environmental stresses that cause DNA damage, and growth and development by supporting DNA replication and transcription [46,47,48]. For example, Guo et al. [47] identified a gene encoding an ATP-dependent DNA helicase associated with plant height on bread wheat chromosome 7B.

Durum wheat’s *TRITD7Bv1G059650* gene encodes a protein with a U-box domain. U-box domain-containing proteins play a crucial role in the ubiquitin-proteasome system (UPS), which is involved in various cellular functions, including stress responses, protein quality control, and signal transduction [49,50,51]. Although the functional characterization of the *TRITD7Bv1G059650* gene in durum has not been conducted, its classification as a U-box domain-containing protein suggests it likely participates in ubiquitination-related processes. The *TRITD4Av1G018270* gene encodes a protein similar to retinoblastoma-binding protein 5 (RBBP5), which is a component of the MLL/SET1 histone H3K4 methyltransferase complex. RBBP5 orthologues are conserved across various species, including cereals, and play a critical role in regulating gene expression through histone modification, particularly the methylation of histone H3 at lysine 4 (H3K4), which is associated with active transcription during development and stress responses [52,53].

In this study, almost all cultivars and breeding populations had stop codons, whereas over 92% of genotypes in the landrace accessions lacked the stop codons in the *TRITD2Av1G282370*, *TRITD7Av1G274730*, and *TRITD7Bv1G059650* genes at a corresponding position described in Table 2. In the case of the *TRITD4Av1G018270* gene (RBBP5 ortholog), all cultivars and breeding populations, as well as 75.3% of landrace genotypes, had a stop codon at the specified position, suggesting selection of the gene during breeding. While direct phenotypic validation is required, this pattern aligns with the gene’s role in the epigenetic regulation of developmental plasticity and breeders’ emphasis on stress-adaptive traits. The retained allele lacking the stop codon in 24.7% of landraces likely reflects standing variation not subjected to artificial selection. This mutation may influence chromatin-mediated gene regulation networks underlying agronomic traits, though functional studies are needed to confirm its phenotypic effects.

*TRITD4Bv1G190600* encodes Tetratricopeptide repeat protein 7A (TTC7A) which is part of a family of proteins with TPR motifs that participate in a variety of cellular processes. The specific function of this gene on wheat chromosome 4B has not been well-defined but could potentially be related to cellular processes such as stress responses and growth regulation. In bread wheat, the tetratricopeptide repeat-TraesCS6B03G1214400 (*TaTPR-B1*) gene on chromosome 6B was reported to regulate spike compactness, which is associated with grain yield [54]. The gene *TRITD2Bv1G204550* encodes a protein belonging to the Basic Helix-Loop-Helix (bHLH) DNA-binding superfamily. The bHLH family is a large group of transcription factors that play key roles in regulating the expression of genes involved in various biological processes, such as cell differentiation, and response to environmental stimuli [55,56,57]. An analysis of the bHLH transcription factor family across the bread wheat genome conducted by Wang et al. [58] revealed their involvement in response to biotic and abiotic stresses. Hence, it is likely that this gene plays a role in transcriptional regulation, including response to environmental stimuli in durum wheat.

The *TRITD2Bv1G223490* gene in durum wheat codes for a protein belonging to the NBS-LRR (Nucleotide-Binding Site-Leucine-Rich Repeat) family. This family of proteins is well-known for its role in plant disease resistance, and this gene is likely involved in resistance to diseases such as rust and fusarium head blight in wheat [59,60,61,62]. The *TRITD6Bv1G052050* gene in durum wheat codes for a calmodulin-binding protein-like protein. These proteins play an important role in calcium signaling in plants, including wheat, which is critical for responding to environmental stimuli, developmental cues, and stress. They are involved in processes such as growth, development, stress responses (e.g., drought, salinity, and pathogen attack), and regulation of enzyme activities [63,64,65]. Based on its orthologues’ functions in other cereals, it is likely that *TRITD6Bv1G052050* plays a role in stress responses, metabolism, or development in durum wheat.

In this study, over 91% of genotypes in the landrace accessions had stop codons while almost all cultivars and breeding populations lack stop codons in the corresponding positions in the *TRITD4Bv1G190600*, *TRITD2Bv1G204550*, *TRITD2Bv1G223490*, and *TRITD6Bv1G052050* genes. This could suggest that the loss of stop codons leads to the synthesis of corresponding proteins that contribute to desirable traits targeted by breeders [66,67].

### 3.3. Genetic Variation Between and Within Accessions and Their Groups

The study reveals moderate genetic diversity among durum wheat accessions, typical of a self-pollinating crop shaped by domestication and breeding. Similar patterns were reported by [68], who noted that modern durum wheat breeding has maintained genetic diversity despite selection bottlenecks, particularly in landraces and breeding populations with diverse founder lines. The low mean effective alleles (Ne = 1.05), Shannon’s index (I = 0.04), and observed heterozygosity (Ho = 0.001) reflect expected homozygosity in cultivated durum wheat, where selection and inbreeding reduce variation [5,9,68]. However, accession 31248 emerged as a robust genetic outlier, exhibiting higher diversity (Ne = 1.42, He = 0.22), elevated average pairwise differences, and a Tajima’s D of 2.21, a pattern inconsistent with technical artifacts like seed mixture. Together with its 21.5% private alleles, these findings suggest that it may retain ancestral diversity or harbor introgressions from breeding programs [26,69]. The fixation index (F) extremes (−1.0 to 1.0) highlight divergent breeding histories; fourteen accessions were fully homozygous (F = 1.0), likely representing pure lines or inbred cultivars, while negative F values (e.g., 33523, F = −1.0) could indicate artificial hybridization or residual heterozygosity.

The low mean polymorphic loci (%PL = 7.9%) underscores genetic uniformity in modern cultivars, though landraces like 31248 (%PL = 49.1%) preserved higher diversity, consistent with their heterogeneous nature [68,70]. Tajima’s D results further differentiate selection pressures; negative values (e.g., 33523, D = −2.12, *p* < 0.001) suggest directional selection in breeding, while positive values (e.g., 31252, D = 2.55, *p* < 0.05) may reflect balancing selection in landraces or breeding lines retaining ancestral diversity [39].

### 3.4. Geographic and Agronomic Group Comparisons

Improved cultivars exhibited higher expected heterozygosity (He = 0.05) and %PL (14.3%) than landraces, likely due to deliberate crosses in breeding programs. Landraces from mid-altitudes (2000–2500 masl) showed elevated diversity (He = 0.040, %PL = 10%), possibly because Ethiopian highlands are a secondary center of durum wheat diversity, where farmers maintain heterogeneous landraces [26]. The Oromia region’s higher private allele count (NLPA = 7) hints at localized adaptation or limited gene flow. Notably, very dense-spike accessions had more private alleles (NLPA = 66), potentially linking spike architecture to unique genetic backgrounds selected during domestication [71].

This study underscores the need to optimize genotyping strategies according to accession heterogeneity levels, particularly when working with mixed panels of pure-line cultivars and genetically diverse landraces. Single-individual genotyping is sufficient for homogeneous accessions (e.g., F = 1.0, Ho ≈ 0) as these typically represent inbred lines or fixed landraces with minimal residual variation. In contrast, heterogeneous landraces (e.g., Ho > 0, He > 0.1, or high %PL) require genotyping of 5–10 individuals per accession to adequately capture within-accession diversity (e.g., 31248, which had 21.5% private alleles). This approach minimizes allele frequency misrepresentation in subsequent GWAS analyses [72], with pooled DNA sequencing serving as a cost-effective alternative for allele frequency estimation [73].

To integrate divergent accession types into a unified GWAS panel, both population structure and heterogeneity within an accession should be considered. As a first step, separate linkage disequilibrium (LD) decay analyses should be conducted for landraces and cultivars to evaluate their compatibility concerning their LD pattern. When significant differences exist, a stratified analysis approach is recommended: genotyping single plants of pure-line cultivars alongside 5–10 plants per landrace accession, followed by separate GWAS for each group and subsequent meta-analysis to integrate results [74,75]. When LD patterns are conserved, a joint GWAS analysis can be performed using linear mixed models (LMMs) that adjust for population stratification via kinship matrices or PCA. Furthermore, random effects at the accession level explain unexplained genetic variance [76,77]. Using this approach maximizes the power to detect marker–trait associations while minimizing spurious signals from population structure.

AMOVA revealed substantial genetic differentiation within the durum wheat panel, with 80.1% of the variation occurring among accessions compared to 19.3% within accessions. The high population structure (AMOVA: 80.1% among accessions; F_ST_ = 0.97) is consistent with expectations for self-pollinating crops such as durum wheat, where restricted gene flow and selection pressures are known to promote genetic divergence [5,68]. While artificial selection and demographic bottlenecks likely contribute to this divergence, the detected loci under selection (e.g., stop codon variants) suggests that local adaptation, possibly to agro-climatic conditions, has further shaped genetic variation. Similar patterns have been reported in other durum wheat panels [27], reinforcing that both neutral and selective processes underlie the observed population structure. Our analysis identified several high-impact loci under selection with putative roles in key agronomic traits. Notably, genes involved in stress response mechanisms predominated, including *TRITD2Bv1G223490* (NBS-LRR family), likely contributing to disease resistance against wheat pathogens [59,60,61,62], and *TRITD6Bv1G052050* (calmodulin-binding protein), potentially mediating abiotic stress tolerance [63,64,65]. The strong differentiation between landraces and improved germplasm in these loci (Table 2) suggests that breeders have consistently targeted these genomic regions, in line with findings from bread wheat studies, where orthologs of these genes were associated with yield (TaTPR-B1 for spike architecture [54]) and stress adaptation (bHLH factors [58]). While functional validation is needed, the convergence between the selection signals identified in this study and known QTLs for stress tolerance and yield-related traits in wheat supports the biological relevance of these loci for durum wheat improvement.

Hierarchical AMOVA revealed significant genetic differentiation among improvement status groups (landraces vs. cultivars vs. breeding populations; F_CT_ = 0.44). This aligns with previous reports of genetic divergence between landraces and improved lines in Ethiopian durum wheat [78]. Interestingly, unlike patterns observed in Ethiopian barley landraces, where altitudinal gradients strongly influenced population structure [79], our study detected no significant geographic or altitudinal genetic structuring. This suggests that historical seed exchange and selection practices in durum wheat cultivation may have attenuated local adaptation signatures evident in other cereal systems. F_ST_ values (mean = 0.80) revealed strong genetic differentiation among landraces, with some pairs approaching fixation (F_ST_ = 1.0), likely due to prolonged isolation and local adaptation. Accession 31,248 displayed exceptionally high genetic diversity (highest pairwise differences: 2306.0), resembling ‘diversity hotspots’ documented in wheat landraces, where heterogeneous selection pressures drive elevated variation in certain accessions [69,80]. Breeding populations showed the highest mean F_ST_ (0.93), consistent with genetic bottlenecks during cultivar development. The broad range of Nei’s distances between accessions (2.7–2497.7) underscores the suitability of this panel for association mapping, particularly given the low within-accession heterozygosity (F_IS_ = 0.80). While we detected no significant genetic differentiation along altitudinal gradients (F_CT_ = −0.05, *p* = 0.93) or among geographic regions (F_CT_ = −0.038, *p* = 0.93), improvement status emerged as the primary factor structuring genetic variation in Ethiopian durum wheat. Although the lack of altitude and region effects should be interpreted cautiously, these patterns suggest that breeding history may have a stronger influence than geographic or agroecological factors in shaping genetic architecture. These findings have important implications for conservation and breeding strategies, though additional studies could further elucidate potential environmental influences on the crop’s genetic variation.

Neighbor-joining cluster analysis revealed distinct genetic clustering among the 376 durum wheat genotypes, with six major and seven minor clusters emerging. The clear separation of breeding populations and cultivars (e.g., Cluster I’s exclusive composition of improved materials) from most landraces aligns with findings by Maccaferri et al. [5] and Mazzucchelli et al. [68], who documented strong population structure in durum wheat due to breeding bottlenecks and the maintenance of divergent allele frequencies between landraces and modern cultivars. However, the presence of landrace accessions 31326 and 33244 within predominantly improved-material clusters (Clusters II and VIII) suggests that these landraces may represent transitional genotypes or harbor introgressions from breeding programs, as observed in Ethiopian wheat gene pools by Mengistu et al. [26]. The extensive admixture of landrace genotypes across Clusters XII and XIII, corresponds to the high within-accession diversity previously reported [26,65]. Notably, the absence of clear geographical clustering among landraces deviates from typical isolation-by-distance patterns, reflecting historical seed exchange, consistent with findings by Mengistu et al. [26]. Frequent seed exchange through informal and commercial networks homogenizes genetic diversity across regions, eroding localized variation. Shared agroecological pressures may further obscure geographic distinctions through parallel selection, while widespread adoption of improved varieties dilutes historical population structure via gene flow between them and landraces. Overall, these anthropogenic and environmental factors have likely reshaped durum wheat’s genetic architecture, contrasting with isolated systems where clear geographic signals persist.

The results of the principal coordinate analysis confirmed the patterns observed by cluster analysis, with PCo1 (explaining 38.1% of the total variance) separating improved materials from landraces, consistent with the high F_ST_ values (0.84–0.94) between these groups. The distinct positioning of accessions 31292, 31326, and 33244 within the improved-material cluster suggests that these may represent ‘elite landraces’ with breeding value, as observed in studies of durum wheat landrace diversity [5]. STRUCTURE analysis (K = 2) confirmed this dichotomy, with most landraces forming a homogeneous blue cluster while improved materials dominated the orange cluster. The admixed ancestry of accessions such as 31248 and 31,269 aligns with established patterns of allele frequency gradients in structured populations [72]. Notably, accession 31248 harbors the largest number of private alleles (1685), followed by 33244 (48 private alleles), suggesting their exceptional potential to introduce novel genetic variation. These findings underscore both the preservation of distinct genetic identities within Ethiopian durum wheat and the values of outlier landraces (e.g., 31248, 33244) as potential bridges for introgression between divergent gene pools in breeding programs. While genomic evidence supports their bridging role, we were unable to assess phenotypic clustering patterns due to insufficient phenotypic data. Future studies integrating detailed phenotyping with genomic data will be essential to evaluate their potential for facilitating beneficial gene flow in breeding populations.

## 4. Materials and Methods

### 4.1. Plant Material

A total of 148 durum wheat accessions comprising 54 landraces, 26 improved cultivars, and 68 breeding populations were used in this study (Supplementary Table S1). The landraces were obtained from the Ethiopian Biodiversity Institute (EBI). The cultivars and breeding populations were obtained from the Debrezeit Agricultural Research Center of the Ethiopian Institute of Agricultural Research (EIAR). The cultivars were locally released in Ethiopia, while the breeding populations were initially received from the International Maize and Wheat Improvement Center (CIMMYT) and are being further bred by EIAR (Supplementary Table S1). To maximize insights into both within-accession and population-level diversity, we employed a stratified sampling approach; fifty-seven accessions (47 landraces, 4 cultivars, and 6 breeding populations) were represented by five genotypes each to assess within-accession variation, with particular emphasis on heterogeneous landraces from diverse agroecologies and breeding lines showing phenotypic variability. The remaining 91 accessions (7 landraces, 22 cultivars, and 62 breeding populations) were represented by single genotypes to expand geographic and improvement-status coverage. This approach enabled the genetic analysis of 376 individuals representing 148 accessions while optimizing resource allocation.

### 4.2. Planting, Leaf Tissue Sampling, and DNA Extraction

Three randomly chosen seeds were planted in a greenhouse at the Swedish University of Agricultural Sciences (SLU, Alnarp, Sweden) in 2 L pots filled with soil for the 91 accessions represented by one plant each. Additionally, 10 seeds per accession were planted in 5 L pots filled with soil for the 57 accessions represented by 5 plants each. After germination, one and five seedlings per accession were maintained for the 91 and 57 accessions, respectively, by removing extra seedlings. Two weeks after planting, ten 6 mm leaf disks from each seedling were sampled separately, collected in each deep well of 96-well plates, and freeze-dried using a CoolSafe ScanVAC Freeze Dryer (LaboGene, Denmark) following Trait Genetics’s recommendation. The plates containing 376 freeze-dried samples were shipped to TraitGenetics (GmbH, Gatersleben, Germany) for high-quality genomic DNA extraction and subsequent genotyping. Genomic DNA was extracted from leaf samples in the TraitGenetics laboratory using a standard cetyltrimethylammonium bromide (CTAB) protocol.

### 4.3. SNP Genotyping and Genotype Data Filtering

An Illumina Infinium 25K wheat single-nucleotide polymorphism (SNP) array was used to genotype the 376 genotypes. The SNP array description can be found at https://www.traitgenetics.com/index.php/service-products (accessed on 3 July 2025). A specific durum wheat cluster file developed by TraitGenetics that differentiates durum wheat from bread wheat was used to score markers on the A and B genomes accurately. Upon obtaining genotypic data, loci with missing data and monomorphic loci were filtered out. This resulted in 7842 polymorphic SNP markers with no missing data, which were used for downstream analysis.

### 4.4. Data Analysis

DnaSP version 6 [81] was used to analyze the site frequency spectra of the accessions by grouping them into different populations. The 57 accessions with 5 genotypes each were grouped into 12 populations, with 11 populations containing 25 genotypes (five accessions) and the 12th population containing 10 genotypes (two accessions). The 91 accessions were grouped into three populations of 30, 30, and 31 genotypes (Figure 1).

The nucleotide diversity [82] and Tajima’s D [39] were calculated using the PopGenome package [83] in R 4.0.0 software (R Core Team, 2020) to reveal the genome-wide variation pattern using a sliding window approach [81]. For this, a sliding window size of 1 Mb and a step size of 200 kb was used, as described in Mulugeta et al. [33]. Genetic diversity parameters, such as the effective number of alleles, Shannon’s Information Index, observed and expected heterozygosity, fixation indices, number of private alleles, and Nei’s unbiased genetic distance were computed using GenALEX v.6.5 [84]. The SNP loci polymorphism information content (PIC) was calculated using Power Marker software 3.25 [85].

The variance within and between accessions and among groups of accessions grouped based on different criteria was determined via analysis of molecular variance (AMOVA) using Arlequin version 3.5.2.2 [86]. Arlequin was also used to estimate pairwise genetic differentiation between accessions and groups and to detect outlier SNP markers through a non-hierarchical finite island model. The significance of the differentiation between accessions and groups was tested with ten thousand permutations. Heatmaps of pairwise F_ST_ and average pairwise differences between and within accessions were generated using R scripts within Rcmd, a console version of the R statistical package linked to Arlequin version 3.5.2.2.

Loci under selection were also determined using Arlequin version 3.5.2.2 based on the joint distribution of population differentiation (F_ST_) and heterozygosity ((heterozygosity within populations)/(1–F_ST_)). For this, a null distribution was assumed under the non-hierarchical finite island model, with 100,000 simulations and 100 demes per population, as described in Excoffier and Lischer [86]. Further analysis of the loci under selection (F_ST_ *p*-value < 0.001) was conducted using Ensembl Plants’ Variant Effect Predictor (VEP) based on the durum wheat reference genome: *Triticum turgidum* (Svevo.v1) at https://plants.ensembl.org/Triticum_turgidum/Info/Index) (accessed on 3 July 2025). Through this analysis, the positions of SNP variants and their effects on associated genes were determined (Supplementary Table S2).

Nei’s unbiased genetic distance-based neighbor-joining cluster analysis, including bootstrap branch support, was conducted using MEGA11 [87], and the resulting trees were viewed and edited with iTOL v5 [88]. Principal coordinate analysis (PCoA) was performed based on Nei’s unbiased genetic distance using GenAlEX6.503 [84].

A Bayesian statistics-based clustering algorithm implemented in the software STRUCTURE version 2.3.4 [30] was utilized to determine the optimal number of genetic populations representing 376 genotypes from 148 accessions. The ADMIXTURE model and correlated allele frequencies were assumed to assess the ancestry fractions associated with each genotype. A burn-in period of 100,000 and Markov Chain Monte Carlo (MCMC) iterations of 200,000 were set for K ranging from 2 to 10 (with 10 independent runs for each K). The STRUCTURE output was then used to determine the optimum K with STRUCTURESELECTOR [31] following the ΔK approach [32]. A beta version of CLUMPACK [89] integrated into the STRUCTURESELECTOR was used to visualize the population structure for the optimal K.

## 5. Conclusions

This study provides valuable insights into breeding for trait improvement, genetic resource conservation, and genotyping strategies in durum wheat. The analysis of genetic diversity and population structure revealed moderate diversity across accessions, with landraces exhibiting higher variation than improved cultivars, making them crucial for trait enhancement through targeted breeding. Key genes under selection (e.g., *TRITD7Bv1G059650*, *TRITD2Bv1G223490*) influence stress tolerance, disease resistance, and yield-related traits, providing targets for marker-assisted breeding. While these candidate genes require further functional validation, their predicted roles align with prior wheat studies, offering testable hypotheses for future research. Adding these loci to a marker set for GWAS in well-characterized panels could further resolve their agronomic relevance. Landraces, such as accession 31248, with high private alleles and diversity, should be prioritized for conservation to maintain the crop’s adaptive potential. The lack of strong geographic structuring suggests that conservation strategies should focus on genetic rather than geographic diversity. Furthermore, this study highlights the need for tailored approaches; single-plant genotyping suffices for homogeneous cultivars, while 5–10 individuals per accession are recommended for heterogeneous landraces to capture within-accession diversity in marker–trait association (MTA) analysis. A stratified approach, accounting for differing LD patterns between landraces and cultivars, enhances MTA analysis accuracy. In summary, integrating genomic insights with optimized genotyping and conservation strategies will accelerate durum wheat improvement while preserving Ethiopia’s unique genetic resources. Future studies should quantify the proportion of landrace diversity retained in modern cultivars to systematically prioritize underutilized genetic resources for breeding.

## Figures and Tables

**Table 1:** The distribution of 6744 SNP markers, across the durum wheat genome, used in the final data analysis.

Chr	NM	GCR (bp)	CGRS (Mbp)	ND	TD (*p* > 0.10)
1A	487	1,159,612–584,771,671	583.612	0.209	1.499
2A	524	295,475–774,814,125	774.519	0.211	1.530
3A	453	304,055–746,465,146	746.161	0.200	1.295
4A	345	698,412–735,809,633	735.111	0.197	1.230
5A	539	27,537–667,289,264	667.262	0.211	1.540
6A	381	770,173–615,260,837	614.491	0.204	1.391
7A	593	173,256–727,310,461	727.137	0.205	1.417
1B	537	313,555–681,099,620	680.786	0.203	1.368
2B	575	406,084–789,416,853	789.376	0.194	1.164
3B	582	306,806–836,443,340	836.137	0.196	1.225
4B	270	1,400,884–675,805,446	674.405	0.221	1.724
5B	563	2,555,603–701,346,725	698.760	0.196	1.217
6B	477	2,064,505–698,590,527	696.526	0.210	1.507
7B	418	113,839–719,907,662	719.794	0.206	1.425
A genome	3322 ^a^	-	4176.5 ^a^	0.21 ^b^	1.41 ^b^
B genome	3422 ^a^	-	5095.8 ^a^	0.20 ^b^	1.38 ^b^
Whole genome	6744 ^a^	-	9272.3 ^a^	0.20 ^b^	1.40 ^b^

Chr = chromosome; NM = number of markers; GCR (bp) = genome coverage range in base pairs; CGRS (Mbp) = covered genomic region size in mega base pairs; ND = nucleotide diversity; TD = Tajima’s D. ^a^ = sum, ^b^ = mean.

**Table 2:** Description of genic loci under selection whose SNPs resulted in a stop codon gain or loss, or amino acid change with moderate to high impacts on their proteins as predicted by Variant Effect Predictor (VEP) tool.

Marker	Chr	SNP Position	Obs. Het.	Obs F_ST_	F_ST_ *p*-Value	SNP	Mutation Type	Impact	AA Change	Codons	SIFT Score	Gene	Gene Description
AX-108886825	2A	749161581	0.33	0.95	0.00245	A/T	Stop-lost	High	*/L	tAa/tTa	-	*TRITD2Av1G282370a*	Exocyst complex component, putative
AX-158531685	6A	99013566	0.31	0.95	0.00419	A/G	missense	Moderate	Q/R	cAg/cGg	0.01	*TRITD6Av1G042660a*	Leucine-rich repeat receptor-like protein kinase family protein
AX-158543425	6B	694134398	0.3	0.98	0.00004	A/C	missense	Moderate	K/Q	Aaa/Caa	0.03	*TRITD6Bv1G227240a*	Seed maturation-like protein
AX-158544944	1B	616467524	0.39	0.68	0.00591	G/T	missense	Moderate	G/W	Ggg/Tgg	0	*TRITD1Bv1G201940a*	ABC transporter B family protein
AX-158554628	7A	713342696	0.33	0.95	0.00245	A/C	Stop-lost	High	*/S	tAg/tCg	-	*TRITD7Av1G274730a*	DNA helicase
AX-94416225	6B	539466257	0.32	0.98	0.00002	C/T	missense	Moderate	R/K	aGa/aAa	0.02	*TRITD6Bv1G168530b*	Enhancer of mRNA-decapping protein 4
AX-94439358	3A	200789462	0.32	0.98	0.00002	C/T	missense	Moderate	T/M	aCg/aTg	0.04	*TRITD3Av1G082030a*	Epoxide hydrolase 2
AX-94458766	3A	481186192	0.32	0.98	0.00002	A/G	missense	Moderate	M/T	aTg/aCg	0	*TRITD3Av1G171840b*	SWAP (Suppressor-of-White-APricot)/surp domain-containing protein
AX-94463985	7B	578955005	0.38	0.94	0.00718	C/T	missense	Moderate	S/N	aGc/aAc	0.04	*TRITD7Bv1G185250b*	Glycosyltransferases
AX-94603856	1B	600672886	0.18	0.63	0.00964	G/T	missense	Moderate	S/I	aGc/aTc	0.01	*TRITD1Bv1G196100a*	60 kDa chaperonin
AX-94639471	2A	51194356	0.36	0.68	0.00952	G/T	missense	Moderate	G/V	gGc/gTc	0	*TRITD2Av1G025670a*	CAP-gly domain linker G
AX-94646444	4B	633082630	0.31	0.95	0.00419	C/T	Stop-gained	High	Q/*	Cag/Tag	-	*TRITD4Bv1G190600a*	Tetratricopeptide repeat protein 7A
AX-94969179	1A	537772342	0.35	0.67	0.00794	C/T	missense	Moderate	P/L	cCc/cTc	0	*TRITD1Av1G206330a*	Pentatricopeptide repeat-containing protein
AX-95006148	2B	611276157	0.32	0.98	0.00002	C/T	Stop-gained	High	Q/*	Cag/Tag	-	*TRITD2Bv1G204550a*	Basic Helix-Loop-Helix (bHLH) DNA-binding superfamily protein G
AX-95073999	2B	674106642	0.32	0.98	0.00002	C/T	Stop-gained	High	Q/*	Caa/Taa	-	*TRITD2Bv1G223490a*	NBS-LRR disease resistance protein-like protein
BS00009789_51	5B	410632320	0.32	0.98	0.00002	G/T	missense	Moderate	P/Q	cCg/cAg	0	*TRITD5Bv1G137170b*	Processing peptidase
BS00046963_51	6B	145924311	0.34	0.98	0.00005	A/C	Stop-lost	High	*/E	Tag/Gag	-	*TRITD6Bv1G052050* *b*	Plant calmodulin-binding protein-like protein
CAP8_c2210_103	6B	679394894	0.2	0.58	0.00004	C/T	missense	Moderate	V/I	Gtc/Atc	0.02	*TRITD6Bv1G221320b*	DNL-type zinc finger protein
Excalibur_rep_c111629_239	7B	538297853	0.25	0.64	0.00217	A/C	missense	Moderate	K/N	aaA/aaC	0.03	*TRITD7Bv1G170020a*	ATP-citrate synthase, putative
Ra_c56305_1946	7B	168560355	0.3	0.98	0.00004	C/T	Stop-gained	High	Q/*	Caa/Taa	-	*TRITD7Bv1G059650a*	U-box domain-containing family protein
RAC875_c65710_156	6B	679642547	0.23	0.61	0.0011	C/T	missense	Moderate	P/L	cCg/cTg	0.02	*TRITD6Bv1G221530a*	n/a
Tdurum_contig15512_429	2B	138766219	0.28	0.98	0.00032	A/G	missense	Moderate	V/A	gTa/gCa	0.03	*TRITD2Bv1G053850b*	Dihydrolipoamide acetyltransferase component of pyruvate dehydrogenase complex
Tdurum_contig97611_150	6A	6813755	0.29	0.96	0.00401	A/G	missense	Moderate	V/A	gTg/gCg	0	*TRITD6Av1G002940b*	Glycosyltransferase
wsnp_Ex_c12818_20334501	4A	101554190	0.32	0.98	0.00002	G/T	missense	Moderate	P/Q	cCa/cAa	0	*TRITD4Av1G042900b*	Serine/arginine repetitive matrix protein 1 G
wsnp_Ex_c55245_57821568	4A	40245812	0.29	0.63	0.00018	A/C	Stop-gained	High	L/*	tTa/tGa	-	*TRITD4Av1G018270b*	Retinoblastoma-binding protein 5
wsnp_Ku_c3081_5776947	4A	588463325	0.32	0.98	0.00002	C/T	missense	Moderate	R/C	Cgc/Tgc	0.01	*TRITD4Av1G201710a*	DWNN domain, A CCHC-type zinc finger protein

Chr = chromosome; AA = amino acid; SIFT = Sorting Intolerant from Tolerant.

**Table 3:** Estimates of different genetic diversity parameters for 57 durum wheat accessions with five genotypes each determined based on 7842 SNP markers.

Accession	Na	Ne	I	Ho	He	uHe	F	%PL	NLPA	%LPA	MFPA	ND	TD
31132	1.00	1.00	0.00	0.00	0.00	0.00	−1.00	0.01	10	0.13	1.00	0.00	1.46
31137	1.00	1.00	0.00	0.00	0.00	0.00	−0.75	0.04	0	0.00	na	0.00	1.38
31141	1.00	1.00	0.00	0.00	0.00	0.00	−1.00	0.03	0	0.00	na	0.00	1.84
31146	1.11	1.10	0.07	0.00	0.05	0.06	1.00	10.65	1	0.01	0.40	0.06	2.55 **
31151	1.00	1.00	0.00	0.00	0.00	0.00	−1.00	0.01	0	0.00	na	0.00	1.46
31158	1.00	1.00	0.00	0.00	0.00	0.00	na	0.00	0	0.00	na	0.00	0.00
31170	1.00	1.00	0.00	0.00	0.00	0.00	−1.00	0.01	2	0.03	1.00	0.00	1.46
31176	1.17	1.12	0.10	0.00	0.07	0.07	1.00	16.74	0	0.00	na	0.07	1.24
31209	1.02	1.01	0.01	0.00	0.01	0.01	0.97	2.38	2	0.03	0.80	0.01	−0.003
31215	1.17	1.12	0.10	0.00	0.07	0.07	0.99	16.79	11	0.14	0.38	0.07	1.24
31220	1.00	1.00	0.00	0.00	0.00	0.00	na	0.00	0	0.00	na	0.00	0.00
31230	1.00	1.00	0.00	0.00	0.00	0.00	1.00	0.01	0	0.00	na	0.00	1.30
31237	1.00	1.00	0.00	0.00	0.00	0.00	−0.56	0.03	4	0.05	1.00	0.00	0.22
31239	1.02	1.01	0.01	0.00	0.01	0.01	0.98	1.63	1	0.01	0.20	0.01	0.03
31246	1.10	1.05	0.05	0.00	0.03	0.04	0.99	10.18	1	0.01	0.80	0.04	0.04
31248	1.49	1.42	0.32	0.03	0.22	0.25	0.83	49.07	1685	21.49	0.40	0.25	2.21 *
31250	1.00	1.00	0.00	0.00	0.00	0.00	−1.00	0.01	4	0.05	1.00	0.00	1.46
31252	1.09	1.09	0.06	0.00	0.04	0.05	1.00	9.23	0	0.00	na	0.05	2.55 **
31260	1.13	1.06	0.06	0.00	0.04	0.05	1.00	12.90	0	0.00	na	0.05	0.03
31266	1.00	1.00	0.00	0.00	0.00	0.00	−1.00	0.03	1	0.01	1.00	0.00	1.84
31269	1.26	1.12	0.13	0.00	0.08	0.09	1.00	26.43	17	0.22	0.20	0.09	0.03
31292	1.01	1.01	0.00	0.00	0.00	0.00	0.84	0.78	0	0.00	na	0.00	1.07
31299	1.00	1.00	0.00	0.00	0.00	0.00	−0.70	0.04	3	0.04	1.00	0.00	0.84
31326	1.12	1.06	0.06	0.00	0.04	0.04	0.99	11.91	9	0.11	0.80	0.04	0.06
31334	1.07	1.03	0.04	0.00	0.02	0.03	0.99	7.24	0	0.00	na	0.03	0.04
31356	1.00	1.00	0.00	0.00	0.00	0.00	na	0.00	0	0.00	na	0.00	0.00
31358	1.16	1.14	0.11	0.00	0.08	0.08	1.00	16.48	6	0.08	0.37	0.08	2.27 **
31361	1.00	1.00	0.00	0.00	0.00	0.00	na	0.00	0	0.00	na	0.00	0.00
31368	1.10	1.05	0.05	0.00	0.03	0.03	0.98	9.81	0	0.00	na	0.03	0.02
31372	1.13	1.06	0.07	0.00	0.04	0.05	1.00	13.45	0	0.00	na	0.05	0.02
31609	1.11	1.06	0.06	0.00	0.04	0.04	1.00	10.74	1	0.01	0.20	0.04	0.43
31696	1.00	1.00	0.00	0.00	0.00	0.00	−1.00	0.05	0	0.00	na	0.00	2.19 *
31979	1.20	1.13	0.11	0.00	0.08	0.09	1.00	19.89	4	0.05	0.33	0.09	1.05
33235	1.00	1.00	0.00	0.00	0.00	0.00	−0.11	0.01	0	0.00	na	0.00	−1.11
33239	1.00	1.00	0.00	0.00	0.00	0.00	−0.33	0.05	7	0.09	1.00	0.00	−0.7
33244	1.20	1.12	0.11	0.00	0.07	0.08	0.99	20.42	48	0.61	0.44	0.08	0.68
33283	1.19	1.13	0.11	0.00	0.08	0.08	0.90	19.15	8	0.10	0.35	0.08	1.24
33286	1.16	1.15	0.11	0.00	0.08	0.09	1.00	15.95	1	0.01	0.40	0.09	2.55 **
33296	1.00	1.00	0.00	0.00	0.00	0.00	na	0.00	35	0.45	1.00	0.00	0.00
33496	1.10	1.08	0.06	0.00	0.04	0.05	1.00	9.68	1	0.01	0.40	0.05	2.19 *
33511	1.15	1.07	0.07	0.00	0.05	0.05	0.99	14.61	4	0.05	0.80	0.05	0.02
33517	1.00	1.00	0.00	0.00	0.00	0.00	−1.00	0.08	17	0.22	1.00	0.00	2.36 **
33523	1.04	1.01	0.01	0.01	0.01	0.01	−0.11	4.13	1	0.01	1.00	0.01	−2.12 ***
33555	1.13	1.09	0.08	0.00	0.05	0.06	0.88	13.06	4	0.05	0.35	0.06	1.40
33681	1.16	1.11	0.09	0.00	0.06	0.07	1.00	15.67	7	0.09	0.23	0.07	1.28
33761	1.15	1.11	0.09	0.00	0.06	0.07	1.00	15.44	7	0.09	0.20	0.07	1.21
33840	1.02	1.01	0.01	0.00	0.01	0.01	0.99	1.99	0	0.00	na	0.01	0.34
Bakalcha	1.24	1.16	0.14	0.00	0.09	0.10	0.96	24.09	45	0.57	0.43	0.10	1.12
Denbi	1.05	1.04	0.03	0.00	0.02	0.02	0.97	4.60	0	0.00	na	0.02	1.95
Ginchi	1.14	1.06	0.07	0.00	0.04	0.05	0.99	13.50	1	0.01	0.20	0.05	0.03
Leliso	1.15	1.07	0.08	0.00	0.05	0.05	0.99	15.00	2	0.03	0.40	0.05	0.08
PON19CD_162	1.10	1.05	0.05	0.00	0.03	0.04	0.98	10.46	7	0.09	0.57	0.04	0.05
PON19CD_251	1.00	1.00	0.00	0.00	0.00	0.00	0.14	0.09	6	0.08	0.87	0.00	1.05
PON19CD_262	1.01	1.01	0.01	0.00	0.00	0.00	0.83	1.15	38	0.48	0.99	0.00	0.68
PON19CD_270	1.01	1.00	0.00	0.00	0.00	0.00	0.79	0.51	3	0.04	1.00	0.00	1.03
PON19CD_276	1.01	1.00	0.00	0.00	0.00	0.00	0.63	0.69	25	0.32	1.00	0.00	0.11
PON19CD_311	1.01	1.01	0.01	0.00	0.01	0.01	0.92	1.36	22	0.28	0.97	0.01	1.22
Mean	1.08	1.05	0.04	0.00	0.03	0.03	0.48	7.86	35.98	0.46	0.64	0.03	0.82

Na = number of alleles; Ne = number of effective alleles; I = Shannon information index; Ho = observed heterozygosity; He = expected heterozygosity; uHe = unbiased expected heterozygosity; F = fixation index; PPL = percent polymorphic loci; NLPA = number of loci with private alleles; %LPA = percentage of loci with private alleles; MFPA = mean frequency of private alleles; ND = nucleotide diversity; TD = Tajima’s D. na = not applicable. *, **, *** = significant at 0.05, 0.01, and 0.001 level, respectively. Note: Several non-zero values have been rounded to zero; see Supplementary Table S3 for more accurate values.

**Table 4:** Estimates of different genetic diversity parameters for a group of durum wheat accessions grouped according to their administrative region (Amhara, Oromia, and Tigray), altitude range, accession type (cultivar, landrace, and breeding population), and spike density (lax, dense, and very dense).

Accession Type	Na	Ne	I	Ho	He	uHe	F	%PL	NLPA	%LPA	MFPA	ND	TD
Amhara	1.08	1.05	0.05	0.000	0.030	0.034	0.60	7.9	2	0.02	0.62	0.03	1.06
Oromia	1.08	1.05	0.05	0.000	0.031	0.035	0.45	8.0	7	0.09	0.56	0.03	1.09
Tigray	1.07	1.05	0.05	0.000	0.030	0.033	0.33	7.5	2	0.02	0.37	0.03	0.94
Below 2000 masl	1.06	1.04	0.04	0.000	0.022	0.026	0.97	6	3	0.03	0.58	0.03	0.56
2000–2500 masl	1.09	1.07	0.06	0.000	0.040	0.044	0.65	10	12	0.15	0.50	0.04	1.27
Above 2500 masl	1.08	1.05	0.04	0.000	0.030	0.033	0.32	7	4	0.05	0.56	0.03	1.14
Breeding population	1.02	1.01	0.01	0.000	0.007	0.008	0.72	2.4	17	0.22	0.90	0.01	0.69
Improved cultivar	1.15	1.08	0.08	0.000	0.050	0.055	0.98	14.3	12	0.15	0.34	0.06	0.80
Landrace	1.08	1.05	0.05	0.001	0.031	0.035	0.40	8.0	40	0.52	0.62	0.03	0.84
Dense	1.07	1.04	0.04	0.001	0.024	0.027	0.14	6.5	3	0.03	0.57	0.03	0.88
lax	1.07	1.05	0.04	0.000	0.028	0.031	0.55	7.1	3	0.04	0.66	0.03	0.52
very Dense	1.09	1.06	0.05	0.001	0.034	0.038	0.67	9.0	66	0.84	0.67	0.04	0.64

masl = meter above sea level; Na = number of alleles; Ne = number of effective alleles; I = Shannon information index; Ho = observed heterozygosity; He = expected heterozygosity; uHe = unbiased expected heterozygosity; F = fixation index; PPL = percent polymorphic loci; NLPA = number of loci with private alleles; %LPA = percentage of loci with private alleles; MFPA = mean frequency of private alleles; ND = nucleotide diversity; TD = Tajima’s D.

**Table 5:** Analysis of molecular variance (AMOVA) of durum wheat accessions without grouping and by grouping them according to their altitude range of collection, administrative regions of origin, accession types, and spike density.

Source of Variation	DF	Sum of Squares	Variance Component	%Age of Variation	Fixation Index	*p*-Value
Among accessions	56.0	348,300.4	592.96Va	80.12	F_ST_ = 0.80	Va and F_ST_ < 0.001
AIWA	228.0	66,133.2	142.95Vb	19.32	F_IS_ = 0.97	Vb and F_IS_ < 0.001
Within individuals	285.0	1,182.5	4.15 Vc	0.56	F_IT_ = 0.99	Vc and F_IT_ < 0.001
Total	569.0	415,616.1	740.063			
Among groups ^a^	2.0	6,691.9	−30.83 Va	−4.86	F_ST_ = 0.79	Vc and F_ST_ < 0.001
AAWG	19	103,781.4	533.04 Vb	84.09	F_SC_ = 0.80	Vb and F_SC_ < 0.001
within accessions	198	26,080.9	131.72 Vc	20.78	F_CT_ = −0.05	Va and F_CT_ = 0.930
Total	219	136,554.2	633.93			
Among groups ^b^	2.0	6,621.78	−21.8 Va	−3.80	F_ST_ = 0.77	Vc and F_ST_ < 0.001
AAWG	23	109,557.7	462.93 Vb	80.49	F_SC_ = 0.78	Vb and F_SC_ < 0.001
within accessions	234	31,364.8	134.04 Vc	23.31	F_CT_ = −0.038	Va and F_CT_ = 0.930
Total	259.0	147,544.3	575.14			
Among groups ^c^	2.0	90,639.9	467.87 Va	44.0	F_ST_ = 0.88	Vc and F_ST_ < 0.001
AAWG	54	257,660.4	464.03 Vb	43.65	F_SC_ = 0.78	Vb and F_SC_ < 0.001
within accessions	513	67,315.7	131.22 Vc	12.34	F_CT_ = 0.44	Va and F_CT_ < 0.001
Total	569.0	415,616,07	1063.11			
Among groups ^d^	2.0	15,599.9	9.71 Va	1.31	F_ST_ = 0.82	Vc and F_ST_ < 0.001
AAWG	54	332,700.51	602.99 Vb	81.06	F_SC_ = 0.82	Vb and F_SC_ < 0.001
within accessions	513	67,315.7	131.22 Vc	17.64	F_CT_ = 0.01	Va and F_CT_ = 0.192
Total	569.0	415,616,07	743.92			

DF = degrees of freedom; AIWA = among individuals within accessions; AAWG = among accessions with groups. ^a^ = twenty-two landrace accessions were grouped into three altitude groups: <2000 m above sea level (masl), 2000–2500 masl, and >2500 masl. ^b^ = twenty-six landrace accessions were grouped according to their administrative regions of origin (Amhara, Oromia, and Tigray). ^c^ = fifty-seven accessions were grouped into landraces, cultivars, and breeding populations. ^d^ = fifty-seven accessions were grouped according to their spike density (lax, dense, and very dense).

## Data Availability

The genotypic data presented in this study were generated using the Illumina Infinium 25K wheat SNP array, details of which can be found at https://www.traitgenetics.com/index.php/service-products (accessed on 30 June 2025). Upon request, the genotypic data of the 376 genotypes studied can be obtained.

## Supplementary Materials

The following supporting information can be downloaded at https://www.mdpi.com/article/10.3390/ijms26157220/s1.
